# Training-induced cognitive coupling emerges under sham but not active transcranial electrical stimulation in older adults: a triple-blind, randomized, sham-controlled study

**DOI:** 10.1186/s12984-026-02077-5

**Published:** 2026-07-14

**Authors:** Yong Jiang, Xue Guo, Isabella Wistuba, Azadeh Lesani, Ivan Chakalov, Perianen Ramasawmy, Andrea Antal

**Affiliations:** 1 Non-Invasive Brain Stimulation Lab, Department of Neurology, University Medical Center Göttingen, Georg-August University of Göttingen, Robert-Koch Straße 40, 37075 Göttingen, Germany; 2 Faculty of Physical Education and Sport Sciences, University of Tehran, Tehran, Iran; 3 Department of Anesthesiology, University Medical Center Goettingen, Goettingen, Germany

**Keywords:** Brain stimulation, Training, Cognition, Healthy elderly, Aging

## Abstract

**Introduction:**

Non-pharmacological methods including training and low-intensity transcranial electrical stimulation (tES) have demonstrated potential in delaying cognitive decline with aging. This study aims to investigate the efficacy of combining repeated tES targeting the left dorsolateral prefrontal cortex with task training on cognitive function in the healthy elderly.

**Methods:**

In this triple-blind, randomized, sham-controlled study, fifty-five participants received ten 20-min sessions of concurrent task training and stimulation, including anodal transcranial direct current stimulation (tDCS + T), theta transcranial alternating current stimulation (tACS + T), or sham tES (sham + T). The training consisted of cognitive N-back tasks and dynamic balance exercises delivered across sessions. Attention Network Test (ANT) and the Stroop Color–Word Test (SCWT) were administered before and after the intervention, with a four-week follow-up. Group × Time effects were analyzed using linear or generalized linear mixed-effects models, adjusting for age, sex, and baseline cognitive status.

**Result:**

Improved overall reaction time (RT) and executive network efficiency were observed in all groups after intervention in ANT. Participants in the sham + T group showed greater improvements with long-term efficacy across all cue-related conditions, and significantly faster RT than tDCS + T group under the double-cue condition at follow-up was observed (χ^2^ (2) = 6.60, p = 0.03, 95% CI [− 0.11, − 0.02], p = 0.002). All groups demonstrated enhanced SCWT performance post-intervention. A significantly stronger negative correlation between orienting network efficiency score and congruent SCWT performance was observed at the follow-up compared to the baseline in the sham + T group (t = 2.686, p = 0.024). Combining tDCS or tACS with task training did not yield superior benefits over sham stimulation.

**Conclusion:**

The observed cognitive improvements likely resulted from training. The absence of add-on effects of tES suggested a potential cancellation between the stimulation-induced modulation and training-driven neural activation induced by task training. Improvements in SCWT performance appear to rely less on orienting or executive engagement after intervention, indicating a shift toward more automatized resource-efficient processing.

*Trial registration*: German Clinical Trials Register, DRKS00031042, Registered June 16, 2023.

**Supplementary Information:**

The online version contains supplementary material available at 10.1186/s12984-026-02077-5.

## Introduction

With age, cognitive functions such as attention and memory gradually decline [1–3]. As the decline progresses, this deterioration may progress to neurodegenerative diseases like Alzheimer’s disease—not only interfering with daily activities and overall well-being in older adults, but also imposing a burden on families, public health systems, and society [1, 4].

Current evidence suggests that the most affected cognitive domains during aging are those related to the prefrontal cortex [5, 6] such as attention [7], working memory [8], information processing [9], and executive function [10]. Evidence from a neuroimaging study demonstrated that old adults showed more activation in the prefrontal cortex during the Stroop task when compared to young participants [11]. Similar findings supported the notion that age-related differences in attentional control were compensated by greater frontal cortical activation across the attention-demanding tasks [12, 13]. In addition to compensatory accounts, several alternative theoretical frameworks have been proposed to explain age-related increases in brain activity. Alternative frameworks, including dedifferentiation, the compensation-related utilization of neural circuits hypothesis, and the scaffolding theory of aging and cognition–revised, suggest that such changes may also reflect reduced neural specificity, increased neural effort, or adaptive reorganization of brain networks [14, 15]. Additionally, the decline of working memory with normal aging accounts for the age-related changes in the dorsolateral prefrontal cortex (DLPFC) [16, 17]. Egner et al.[18] further addressed that the activation of DLPFC is correlated with the cognitive control adjustments, while considerable evidences suggested the deterioration of hippocampus with aging also contributes to the cognitive impairment in old adults [19–21], as recent studies revealed its involvement in executive function [22], intelligence [23], and spatial processing [24]. Results from a study which targeted the relationship between information processing, executive function, and the hippocampus volume in aging adults revealed that the hippocampus volume influences the decline in cognitive function, independent of age [22]. It was proposed that the premorbid hippocampal volume in patients with mild cognitive impairment (MCI) is a predictor of their future progression into Alzheimer’s disease [25].

While pharmacological agents have demonstrated minimal benefit to the improvement of cognition ability [26, 27], non-pharmacological approaches based on neuroplastic changes, including task training [28] and transcranial electrical stimulation (tES) [29, 30], have shown efficacy on improving cognition in old adults.

Previous studies implementing structured comprehensive training [31] and aerobic training [32–34] longitudinally found an increase in improved hippocampus volume, consequently improving cognitive function. For example, a 6-month moderate walking training improved executive function in the elderly when compared to the control group [35]. Recent studies suggested cognition function can be also enhanced through increased cognitive-related brain activities and cortical functional connectivity [36], which can be induced by task training such as coordination training [37], dancing training [38], balance training [39], working memory training [37], computerized game training[40], and dual task training [41]. A randomized, controlled trail, which recruited 2802 healthy old participants, reported overall cognitive improvement after 10 sessions of 60-min either memory or reasoning training and can be observed after a two-year follow-up [42].

tES is non-invasive technique that has used to modulate cortical excitability and activity by applying a weak current through the electrodes on the scalp. It primarily includes transcranial direct current stimulation (tDCS) and transcranial alternating current stimulation (tACS), both of which have been reported to improve attention, memory, and executive functions when applied over the left-DLPFC [43–47]. tDCS delivers a constant current as anodal and cathodal stimulation. Rather than directly inducing neuronal firing, anodal tDCS shifts the resting membrane potential and increases the possibility of of spontaneous neuronal firing rates therefore it can enhance cortical excitability[48, 49]. In opposite to that cathodal tDCS decreases the possibility of the neuronal firings [49]. tACS can modulate endogenous brain oscillations by delivering oscillatory currents at specific frequencies. Previous studies have found that tACS induces local neuron entrainment through modulating neural spike-timing [50], resulting in long-lasting after-effect [51]. Above tACS-related neural mechanisms ultimately contributed to affect the information flow and thereby modulating the cognition function [52]. While polarity / frequencies have great impact on the net effects of tES, other factors such as stimulation duration, montage, intensity, and brain state, also contributed to the intervention results [53, 54]. It has been reported that a single session of 2 mA 30-min anodal tDCS of the left-DLPFC improved executive function when compared with right-DLPFC stimulation and sham within healthy young adults [55]. Similar results were reported in MCI patients [56]. Previous review has demonstrated that theta-frequency tACS targeting left-DLPFC improved executive function, attention, and working memory [57, 58].

Consequently, it has been suggested that combining tES with structured training may yield more cognitive improvements and worthy further exploration [59, 60]. The synergistic effect on engaging cognitive training individuals with concurrent stimulation was reported and improved cognitive function in the elderly was identified after intervention [61, 62]. A 12-week 40-h intervention combining tDCS with computerized cognitive training was applied in the healthy elderly improved executive function assessed via Stroop task in the active group when compared with sham [61]. Improved cognitive attention was observed in MCI patients after 8 sessions of 20-min, 1.5 mA (peak-peak) theta-tACS targeting the frontal lobe combined with cognitive control training [63]. Despite the promising results of combined methods, inconsistent findings have been reported [64, 65]. For instance, combined anodal tDCS targeting left-DLPFC with cognitive training for 9 20-min sessions, or with working memory training for 20 40-min sessions, did not improve the working memory performance in the elderly when compared to sham group [64, 66].

Given the inconsistencies among programs, we aimed to evaluate the efficacy of a combined intervention strategy in a clinical trial. In this study, we concurrently applied 10 20-min sessions of anodal tDCS or theta- tACS targeting the left-DLPFC with a cognitive and motor task training in the healthy elderly. Our main objective was to explore if combining tES with task training was more effective in optimizing cognitive performance. Our secondary aim was to investigate the differential stimulation effects that may account for the improvements, given the distinct modulation characteristics of tDCS and tACS. We hypothesized that active stimulation (tACS and tDCS) combined with training would lead to greater improvements in cognitive attention and inhibition compared to sham stimulation. Furthermore, given the frequency-specific properties of theta-tACS, we expected that tACS might show enhanced effects relative to tDCS.

## Methods

### Study design

This randomized, sham-controlled, triple-blinded study was conducted from 2023 to 2025 at the University Medical Center Göttingen, Germany. Participants were randomly allocated to one of three groups: the tDCS and training group (tDCS + T), the tACS and training group (tACS + T), or the sham and training group (sham + T). Sham condition was categorized into sham tACS and sham tDCS. The study was conducted in accordance with the Declaration of Helsinki. Ethical approval was granted by the local ethics committee (ID: 4/7/22) and was registered in the German Clinical Trials Register (DRKS00031042). Written and verbal informed consent was obtained from all participants prior to enrollment.

### Protocol deviations from trial registration

The original trial registration included four study arms, including a training-only group. During the early recruitment phase, before the start of the official experiment in July 2023 and before any outcome data were analyzed, the training-only arm was removed from the final study design. This decision was made because of recruitment feasibility and to preserve the triple-blind design, as including a no-stimulation training-only group would have compromised blinding. The final study therefore included three arms. The present manuscript reports outcomes that were listed as secondary outcomes in the original trial registration. Accordingly, the present manuscript should be interpreted as an analysis of pre-specified secondary outcomes rather than as the primary outcome report of the registered trial. Accuracy was registered as a secondary outcome and is reported accordingly. In the present dataset, however, accuracy showed near-ceiling values with limited variability. Therefore, accuracy was interpreted mainly as a secondary indicator for assessing possible speed–accuracy trade-offs when interpreting reaction time effects. This interpretive role does not change its status as a registered secondary outcome.

All participants followed an identical intervention schedule consisting of ten 20-min sessions of concurrent stimulation and task training delivered over 4 weeks (max. three sessions per week). The intervention was structured such that the first five sessions involved cognitive N-back training, followed by five sessions combined with dynamic balance training. Sessions were scheduled regularly across the intervention period, and no washout period was included, as the study was designed as a continuous combined intervention.

To ensure proper blinding, six stimulators were coded and pre-programmed by an independent researcher who was not involved in data collection or analysis. Two stimulators were assigned to each stimulation condition under the same code but with different identifiers (e.g., A1 and A2 for tACS, B1 and B2 for tDCS, and C1 and C2 for sham stimulation). For the sham condition, both sham tDCS and sham tACS protocols were used to match the respective active stimulation modes, but were combined into a single sham group for analysis. Participants were allocated using a block randomization procedure stratified by sex, age, height, and body weight. In addition to participant and investigator blinding, data analysis was also conducted in a blinded manner (triple-blind design).

### Participants

The required sample size was estimated using G*Power 3.1 (F tests, ANOVA: repeated measures, within–between interaction). Parameters were set as follows: effect size f = 0.25, α = 0.05, statistical power = 0.95, and correlation among repeated measures = 0.50. The analyses indicated a minimum of 18 participants per group.

Local newspapers and flyers were utilized for the recruiting. Healthy older adults aged 60 to 85 years with a Montreal Cognitive Assessment (MoCA) score of 26 or higher were eligible to participate. Exclusion criteria included (1) any acute medical condition requiring hospitalization within the past three months; (2) metal implants in head or pacemakers, ferromagnetic objects, or other objects with risk of contact with the electrodes; (3) use of medications affecting the central nervous system; (4) acute cardiovascular disease such as uncontrolled or untreated high or low blood pressure; (5) presence of lower-extremity pain, musculoskeletal disorders, or any other condition that may influence gait and balance; (6) diagnosis of any major neuropsychiatric disorders including drug/alcohol addiction, (7) visual impairments, e.g., glaucoma.

**Fig. 1:**
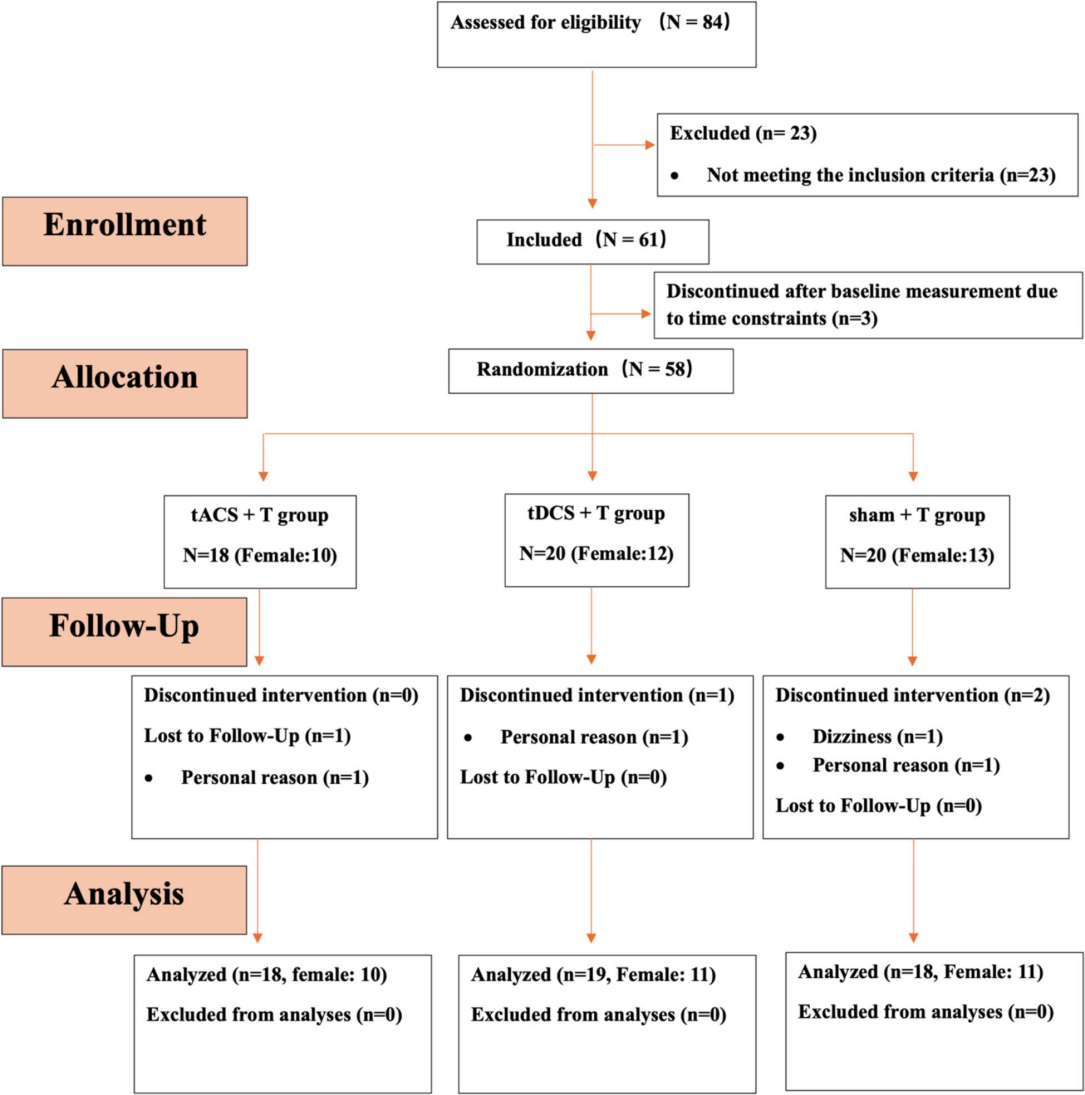
Consolidated Standards of Reporting Trials flow diagram of participants in the study

Fifty-eight participants ranging in age from 60 to 85 years were randomized. Two participants withdrew due to personal reasons, and one individual withdrew because of dizziness after attending two intervention sessions. In total, 55 participants (32 females) were included in the data analysis (Fig. 1).

### Transcranial electrical stimulation

tES was applied with CE-certified battery driven stimulators (NeuroConn direct current stimulator; NeuroConn GmbH, Ilmenau, Germany). Active tACS was delivered at 4 Hz with a peak-to-peak amplitude of 2 mA, while a constant current of 2 mA was applied for tDCS. These stimulation parameters were selected based on commonly used protocols and previous studies, which have been shown to effectively modulate cortical excitability within established safety limits [67, 68]. A pair of 5 × 5 cm conductive rubber electrodes were placed over the left dorsolateral prefrontal cortex (DLPFC) using an F3–Fp2 montage according to the international 10–20 EEG system. The conductive cream (AC cream, GVB geliMed GmbH, Germany) was applied to reach optimal impedance. To minimize the discomfort, there was a 15 s ramp up and 15 s ramp down before / after the stimulation. Sham conditions were programmed for a 30 s stimulation period with a 15 s fade-in/out duration.

Prior to and following each stimulation session, we documented the following symptoms to assess any adverse effects experienced by participants: tingling, headache, dizziness, fatigue, nervousness, and sleep disturbances. Furthermore, skin redness was documented as observed by the stimulation operator. To evaluate potential unblinding effects, participants were requested to indicate their perception of the type of stimulation they had received following the final intervention.

### Task training protocol

**Fig. 2:**
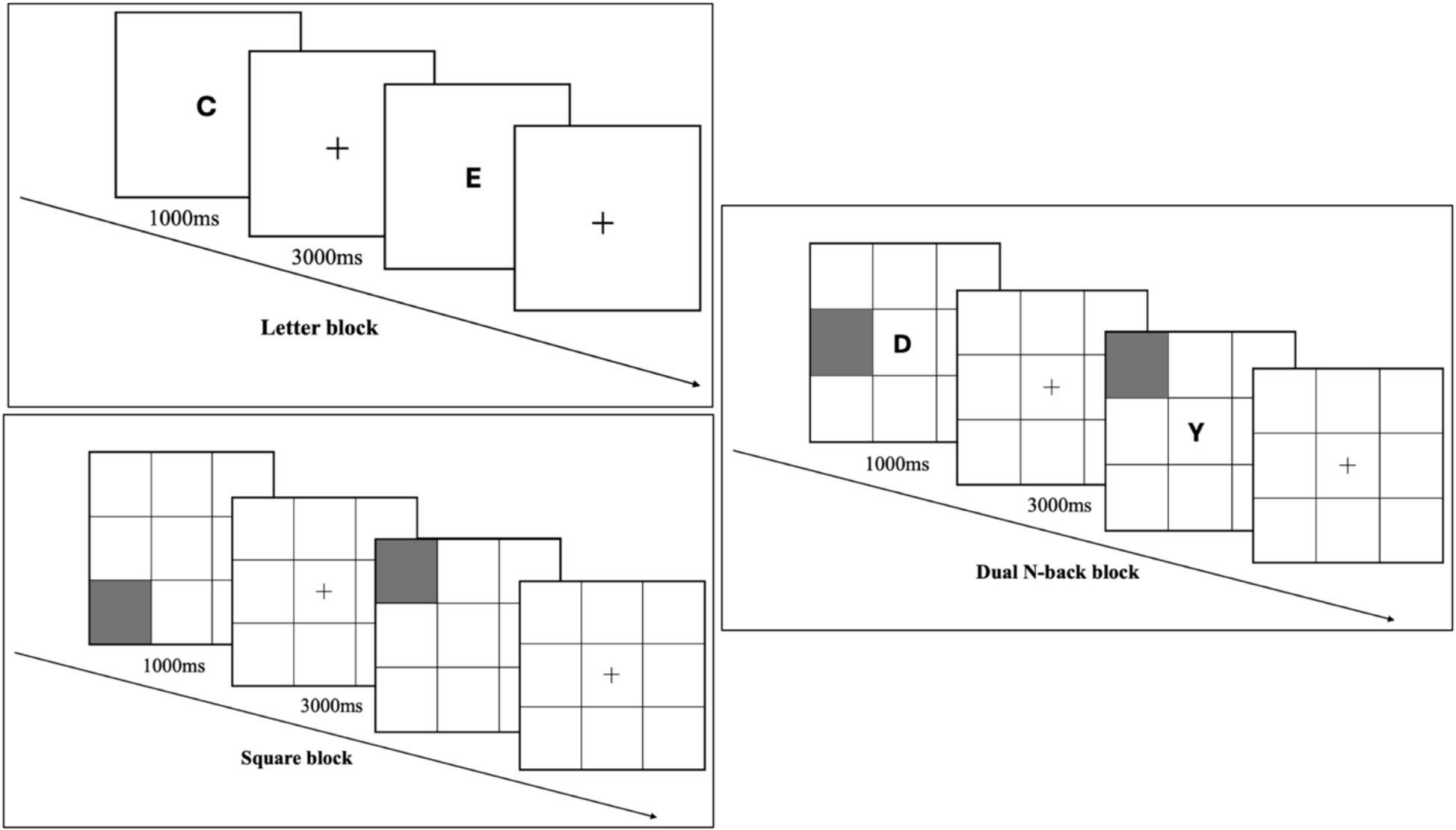
The illustration of N-back training program

Each stimulation session was delivered concurrently with the training tasks. The training task changed over the stimulation days, with five 20-min N-back training sessions followed by five 20-min dynamic balance training sessions. During the cognitive training sessions, participants sat comfortably and completed an N-back task displayed on a 24-inch monitor. The training was administered using PEBL2. Each session consisted of alternating blocks that involved either letters or squares. In the letter blocks, participants pressed the left Shift key under 1-back and 2-back conditions (Fig. 2). Each letter appeared on the screen for 1000 ms, followed by an interstimulus interval ranging from 3000 to 5000 ms, which was adjusted based on performance in the preceding training session. During the square blocks, a nine-square grid appeared at the center of the screen, with a single square randomly shown in one of the eight peripheral locations. Participants responded using the right Shift key in both 1-back and 2-back conditions. Within the dual N-back blocks, letters and squares were presented simultaneously. The letters always appeared in the center of the grid, while the squares occupied the peripheral positions. Here, the left Shift was used for letter matches, and the right Shift for square matches. More specifically, in the 1-back condition, participants were instructed to press the left Shift key only when the current letter matched the previous one but the square appeared in a different position. Conversely, when the square’s position repeated while the letter changed, only the right Shift key was pressed. When both the letter and the square matched their preceding stimuli, participants were required to press both keys simultaneously.

The dynamic balance training program consisted of three parts in each session: a 4-min warm-up, 12 min of balance and standing training, and a 4-min relaxation period. Training sessions were supervised and guided by an experienced physical education instructor.

*Warm-up*: Whole-body stretching and joint mobilization.

*Balance and standing training*: Balance exercises performed either barefoot or using a Bosu ball. Including:

(a) single-/double-leg standing. Participants were standing still on the ground and BOSU ball with single/double legs;

(b) Stair-climbing. Participants began standing on the ground with the BOSU ball positioned directly in front of them. They stepped onto the ball in a stair-like manner, placing one foot at a time on the dome surface, and then stepped backward to return to the ground.

(c) Up-and-Over. Participants stood on the ground with the BOSU ball placed to either the left or right side. They stepped laterally onto the ball and continued moving in the same direction to step off on the opposite side. They then returned to the starting position before repeating the movement.

(d) Standing Scale. Participants stood facing the BOSU ball and stepped onto it with one foot, stabilizing themselves on the dome surface. They extended the contralateral leg backward while simultaneously lifting both arms forward and upward, holding a single-leg “standing scale” posture for several seconds. They then returned to the standing position and repeated the task on the opposite leg. Assistance was provided if required to ensure postural stability.

(e) Cognitive–Motor Stepping. Participants performed two stepping tasks that required simultaneous motor execution and verbal-instruction processing while interacting with a BOSU balance trainer. Task 1: Participants stood on the ground facing the BOSU ball and began by marching in place. In response to verbal cues (“Links Hoch” = left foot up; “Rechts Hoch” = right foot up), they stepped onto the ball with the instructed foot, placed the opposite foot on the ball, and continued marching while maintaining balance on the dome surface. Upon receiving the corresponding step-down command (“Links Unten” = left foot down; “Rechts Unten” = right foot down), participants stepped backward off the ball with the indicated foot, returned both feet to the ground, and resumed marching in place. Task 2: The procedure was identical to Condition 1, except participants were required to respond with the opposite limb to the verbal instruction (e.g., “Links Hoch” = step up with the right foot; “Rechts Unten” = step down with the left foot), introducing an inhibitory and cognitive-control component to the task.

*Relaxation*: Whole-body stretching.

### Outcome measurement

**Fig. 3:**
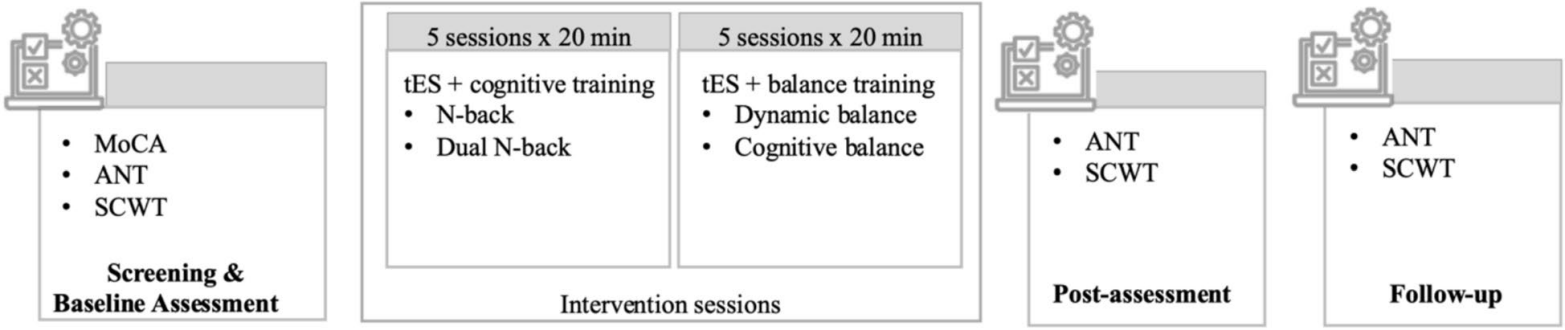
Diagram of study design. Post-assessments were conducted within 24 h after the last intervention. Follow-up assessments were conducted 4-week after the last intervention. MoCA: Montreal Cognitive Assessment; ANT: attention network test; SCWT: Stroop Color-Word Test

Measurements were conducted before the first intervention session (BA), within 24 h after the last intervention session (PA), and four weeks after the last intervention session (FU), as shown in Fig. 3.

### Attention network test

**Fig. 4:**
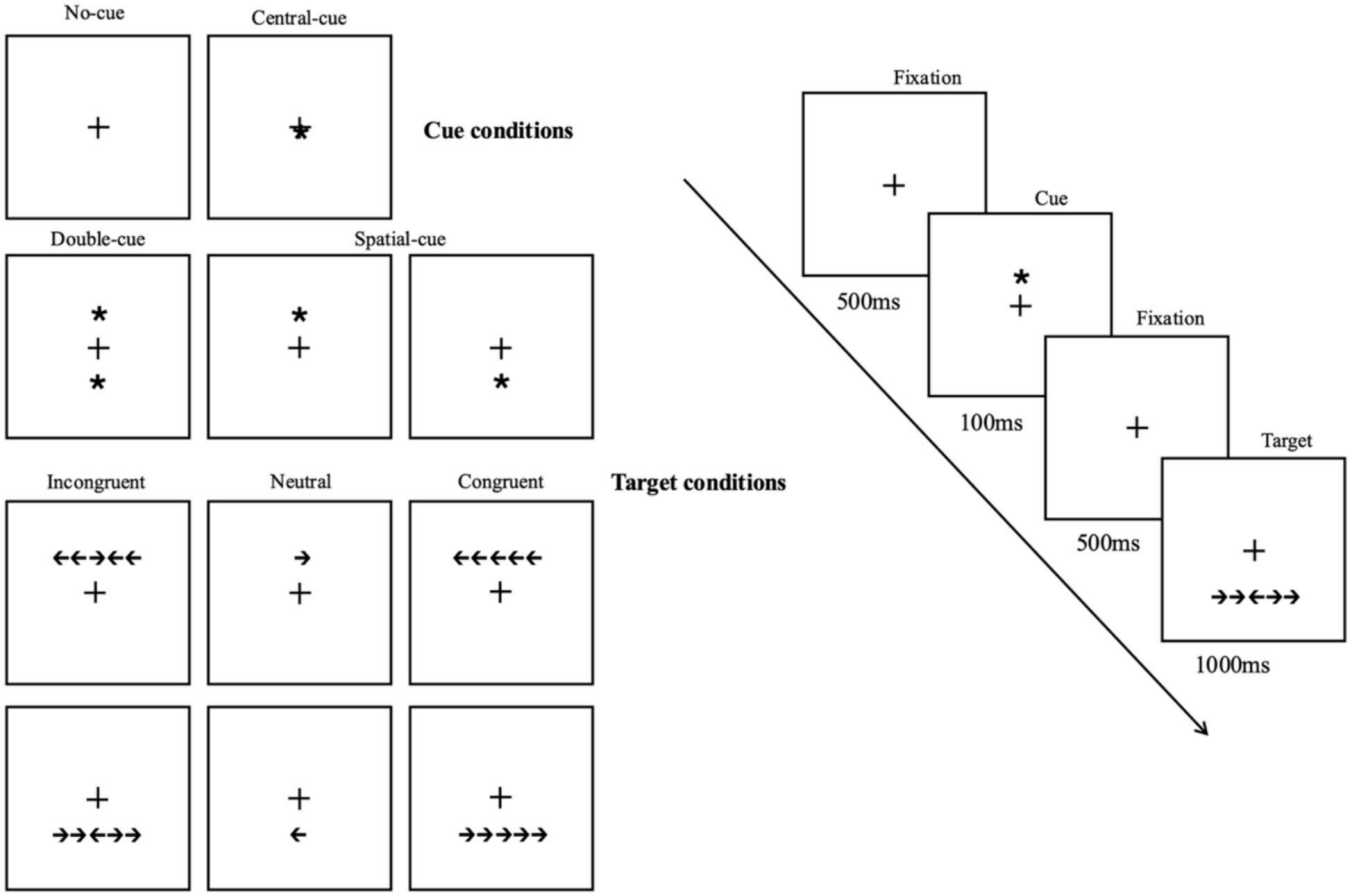
The illustration of ANT

For assessing the network efficiency across the attention processing including alerting, orienting, and executive control, the attention network test (ANT) was applied [69] (Fig. 4). It consisted of cue and target (arrow) conditions. Four cue types were used: (1) no cue, in which no asterisk was presented; (2) center cue, where an asterisk appeared superimposed on the central fixation cross; (3) double cue, with two asterisks displayed simultaneously above and below the cross; and (4) spatial cue, in which an asterisk appeared either above or below the cross to indicate the upcoming target location.

For the target stimuli, three arrow conditions were applied: (1) congruent, in which all five arrows pointed in the same direction; (2) neutral, with only the central arrow presented; and (3) incongruent, in which the central arrow pointed in the opposite direction to the flanking arrows.

Participants were seated and instructed to focus on a fixation cross displayed at the center of the screen. Each trial began with a cue presented for 100 ms, followed by the target arrow, which remained visible for up to 1700 ms. Participants were asked to respond as quickly and accurately as possible by pressing the left or right Shift key to indicate the direction of the central arrow. The reaction time (RT) and accuracy within each condition was documented. For assessing the efficiency, the method described by Mahoney et al. [70] was applied. The mean RTs within different cue and target conditions were documented and the efficiencies were calculated as follows: *Alerting efficiency, reflecting the ability to achieve and maintain an alert state, was calculated as RT_no-cue − RT_center-cue*; *Orienting efficiency, reflecting the ability to selectively shift attention to a spatial location, was calculated as RT_center-cue − RT_spatial-cue*; *Executive control efficiency, reflecting the ability to resolve conflict between competing stimuli, was calculated as RT_incongruent − RT_congruent*.

### Stroop color-word test

To further assess inhibitory control, the Stroop Color-Word Test (SCWT) was administered, which evaluates the ability to suppress cognitive interference arising from competing stimulus attributes [71]. The German version of the SCWT was used. The response keys ‘1’, ‘2’, ‘3’, and ‘4’ corresponded to the colors rot, blau, grün, and gelb (red, blue, green, and yellow, in English respectively). Three trial types were presented: (1) congruent, in which the word’s meaning and its font color were identical (e.g., the word Blau printed in blue, requiring a response with key ‘2’); (2) incongruent, in which the word’s meaning and color were inconsistent (e.g., the word Blau printed in red, requiring key ‘1’); and (3) neutral, in which the word’s meaning was unrelated to any color (e.g., the word WHEN printed in yellow). Each trial began with a fixation cross displayed for 1000 ms, followed by a stimulus presented for up to 3000 ms.

Furthermore, the correlations between attentional network efficiencies and Stroop conditions were investigated.

### Statistical analyses

To ensure baseline comparability among groups, one-way ANOVA was performed on demographic characteristics, including age, MoCA score, height, and weight, after checking homogeneity of variance with Levene’s test. For each dependent variable, normality within each Group × Time cell was assessed through the Shapiro–Wilk test. According to the distributional characteristics of the outcome variables, either linear mixed-effects models (LMMs) or generalized linear mixed-effects models (GLMMs) were employed. Specifically, LMMs were applied when the data met the assumptions of normality, whereas GLMMs were used for non-normally distributed outcomes. For GLMMs, the appropriate distribution family and link function were selected based on the data characteristics. The general analytical model followed the structure: Outcome ~ Group × Time + Age + Sex + MoCA + (1 | Subject), where Group and Time were treated as fixed factors, and random intercepts were assigned to individual participants.

All analyses were conducted in R using the lme4, glmmTMB, and lmerTest packages. Besides, for variables showing marginal or significant group differences at baseline, baseline values were additionally included as covariates in the model (see Supplementary Table S1 for details).

For handling missing data, analyses were conducted both on the original dataset and on datasets processed with several imputation methods, including mean substitution, median imputation, random forest, and predictive mean matching. The model based on the original dataset (with missing values retained) yielded the lowest Akaike Information Criterion [72], indicating superior model fit. Consequently, all statistical analyses were performed using the original data.

When significant main or interaction effects were detected, Bonferroni-adjusted post hoc comparisons were conducted. Effect sizes were reported as partial eta squared (ηp^2^) for LMMs [73], while marginal and conditional R^2^ (R^2^m, R^2^c) were reported for GLMMs.

To assess the associations between attentional network efficiencies and Stroop performance, separate models were fitted with Stroop performance as the outcome variable. Prior to analysis, ANT network efficiencies were mean-centered to facilitate interpretation of interaction terms and to reduce potential multicollinearity arising from higher-order interactions. A three-way interaction model was then applied, including the mean-centered network efficiency, Group, and Time as fixed effects. Post-hoc analyses of simple slopes were conducted when interactions or main effects were significant. Slope estimates, t-value and p-values were reported.

All LMM and GLMM analyses were carried out using R (version 4.5.1) and RStudio, whereas conventional statistical tests were conducted with IBM SPSS Statistics 20.0. The significance level was set at p < 0.05.

## Results

There were no significant differences in participant demographics among groups at baseline, as shown in Table 1.

**Table 1:** Demographics of participants at baseline

Demographics	Sham + T	tACS + T	tDCS + T	P-value
Age, years	71.06 ± 0.68	72.26 ± 0.78	71.94 ± 0.73	0.48
Sex (F/M)	10/8	11/8	11/7	-
Height (cm)	171.39 ± 1.19	169.79 ± 1.26	168.94 ± 1.13	0.35
Body weight (kg)	74.94 ± 1.60	73.21 ± 2.04	71.50 ± 1.45	0.38
MoCA	26.67 ± 0.30	28.05 ± 0.35	27.89 ± 0.34	0.71

Data are presented as mean and standard error of the mean (SE), unless otherwise specified. sham + T: concurrent sham stimulation combined with training group, tACS + T: concurrent tACS combined with training group, tDCS + T: concurrent tDCS combined with training group. MoCA: Montreal Cognitive Assessment test

## Attention network test

### Network efficiency

We first investigated the intervention effects on the efficiency of alerting, orienting, and executive control networks within the ANT.

**Fig. 5:**
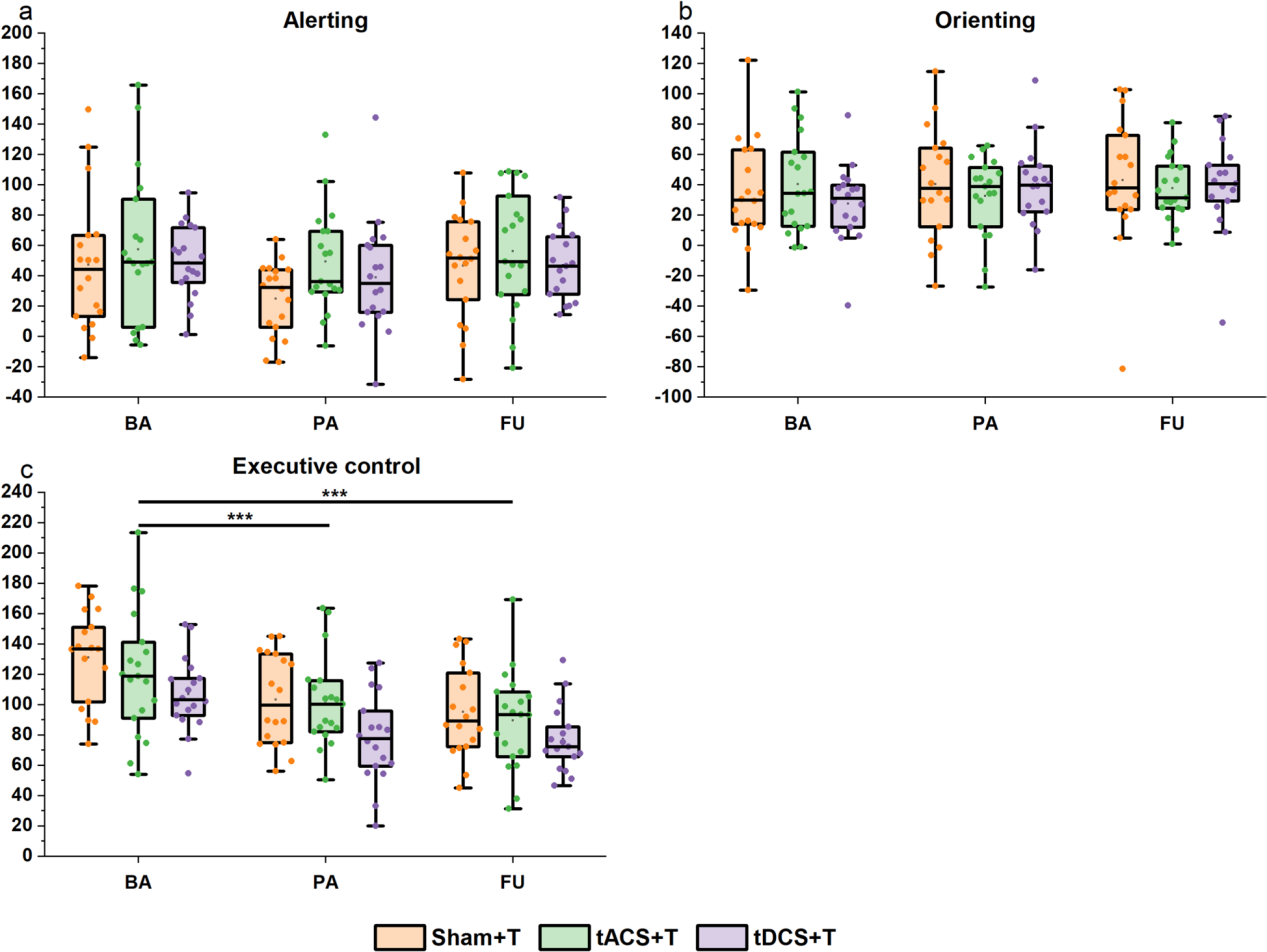
Performance of the three attentional networks. Data are presented as boxplots with overlaid individual participant data points for each group (Sham + T, tACS + T, tDCS + T). The box represents the interquartile range (IQR), the horizontal line indicates the median, and whiskers denote the data range. BA = baseline; PA = post-assessment; FU = follow-up. Panels a–c correspond to the Alerting, Orienting, and Executive control networks, respectively. For the Executive control network, lower scores indicate better efficiency. *: 0.01 < p ≤ 0.05; **: 0.001 < p ≤ 0.01; ***: p ≤ 0.001

No interaction effect was observed in the alerting network. A significant main effect of Time indicated improved efficiency after the intervention (F (2,109.58) = 3.53, p = 0.03); however, post hoc comparisons did not reach statistical significance (95% CI [− 27.41, 0.38], p = 0.06; see Table 2; Fig. 5a). We did not find any alteration in the orienting network (Fig. 5b).

**Table 2:** Performances and results of the attention network test and the stroop color-word test

Parameters	Baseline control ^a^	Sham + T			tACS + T			tDCS + T			Interaction	Group effect	Time effect	R^2^m	R^2^c
		BA	PA	FU	BA	PA	FU	BA	PA	FU					
Alerting	N	47.11 ± 10.55	24.81 ± 5.69	46.58 ± 8.27	57.51 ± 11.22	49.26 ± 7.74	56.09 ± 9.14	48.96 ± 5.73	38.96 ± 8.88	47.07 ± 5.69	F (4, 151) = 0.47, p = 0.75	F (2, 151) = 0.15, p = 0.85	**F (2, 151) = 3.48, p = 0.03**^**b**^**, nP**^**2**^** = 0.38**	–	–
Orienting	N	35.00 ± 8.11	40.38 ± 8.57	43.17 ± 10.08	40.41 ± 7.28	32.75 ± 5.91	37.67 ± 4.70	27.63 ± 6.06	39.40 ± 6.43	39.06 ± 7.57	χ^2^ (4) = 2.56, P = 0.63	χ^2^ (2) = 2.04, p = 0.36	χ^2^ (2) = 0.80, p = 0.67	0.07	0.18
Executive control network	Y	153.26 ± 23.38	103.23 ± 7.09	95.21 ± 7.00	120.14 ± 9.46	101.45 ± 6.82	89.46 ± 7.49	106.72 ± 5.67	77.66 ± 6.92	77.35 ± 5.37	χ^2^ (4) = 2.83, P = 0.58	χ^2^ (2) = 1.77, p = 0.41	**χ**^2^** (2) = 21.15, p < 0.001**	0.48	0.56
No-cue ANT (ms)	N	782.84 ± 25.86	714.44 ± 17.57	721.75 ± 18.70	748.89 ± 23.66	728.43 ± 20.09	726.98 ± 20.68	757.29 ± 21.98	744.89 ± 28.21	739.87 ± 22.27	χ^2^ (4) = 9.30, p = 0.053	χ^2^ (2) = 1.59, p = 0.45	**χ**^2^** (2) = 24.25, p < 0.001**	0.17	0.78
Accuracy	Y	0.96 ± 0.01	0.99 ± 0.003	0.98 ± 0.004	0.98 ± 0.004	0.99 ± 0.002	0.99 ± 0.001	0.99 ± 0.002	0.99 ± 0.005	0.99 ± 0.004	**F (4, 151) = 2.54, p = 0.04, np**^2^** = 0.06**	F (2, 151) = 2.89, p = 0.058	**F (2, 151) = 8.15, p < 0.001, np**^2^** = 0.10**	**–**	**–**
Central-cue ANT (ms)	N	737.98 ± 22.52	687.58 ± 15.95	678.37 ± 16.13	715.64 ± 18.68	690.11 ± 18.00	678.27 ± 18.53	715.90 ± 17.84	700.59 ± 24.80	703.15 ± 23.03	**χ**^2^** (4) = 10.28, p = 0.03**	χ^2^ (2) = 1.02, p = 0.59	**χ**^2^** (2) = 26.13, p < 0.001**	0.17	0.81
Accuracy	Y	0.96 ± 0.02	0.98 ± 0.005	0.99 ± 0.003	0.97 ± 0.005	0.99 ± 0.003	0.99 ± 0.001	0.99 ± 0.002	0.98 ± 0.005	0.99 ± 0.002	F (4, 151) = 1.59, p = 0.18	F (2, 151) = 1.80, p = 0.16	**F (2, 151) = 6.30, p = 0.002, np2 = 0.08**	–	–
Double-cue ANT (ms)	Y	735.71 ± 20.18	685.45 ± 14.36	675.17 ± 17.74	691.38 ± 16.43	679.16 ± 17.06	670.88 ± 17.26	708.32 ± 19.39	705.92 ± 27.23	692.79 ± 20.07	**χ**^2^** (4) = 13.34, p = 0.01**	χ^2^ (2) = 0.22, p = 0.89	**χ**^2^** (2) = 31.02, p < 0.001**	0.74	0.83
Accuracy	N	0.96 ± 0.01	0.98 ± 0.004	0.99 ± 0.004	0.98 ± 0.003	0.98 ± 0.004	0.99 ± 0.002	0.98 ± 0.003	0.98 ± 0.009	0.99 ± 0.003	χ^2^ (4) = 4.40, p = 0.35	χ^2^ (2) = 5.73, p = 0.056	**χ**^2^** (2) = 8.86, p = 0.01**	0.08	0.20
Spatial-cue ANT (ms)	N	704.33 ± 21.50	647.19 ± 15.77	635.19 ± 21.48	673.95 ± 19.27	657.37 ± 18.29	642.19 ± 17.61	688.27 ± 18.54	661.24 ± 28.31	664.08 ± 22.61	**χ**^2^** (4) = 12.75, p = 0.01**	χ^2^ (2) = 1.39, p = 0.49	**χ**^2^** (2) = 35.52, p < 0.001**	0.18	0.82
Accuracy	N	0.97 ± 0.01	0.99 ± 0.005	0.98 ± 0.004	0.98 ± 0.005	0.99 ± 0.002	0.99 ± 0.003	0.99 ± 0.002	0.98 ± 0.007	0.99 ± 0.003	χ^2^ (4) = 3.15, p = 0.53	χ^2^ (2) = 4.28, p = 0.11	χ^2^ (2) = 5.75, p = 0.056	0.06	0.21
Congruent SCWT (ms)	N	1174.92 ± 62.23	1048.71 ± 63.58	968.12 ± 67.97	1091.12 ± 52.18	991.14 ± 46.04	931.28 ± 42.43	1146.48 ± 80.38	1098.02 ± 73.08	996.89 ± 53.58	χ^2^ (4) = 5.08, p = 0.27	χ^2^ (2) = 1.31, p = 0.51	**χ**^2^** (2) = 45.13, p < 0.001**	0.28	0.85
Accuracy	N	0.96 ± 0.007	0.97 ± 0.006	0.98 ± 0.005	0.95 ± 0.01	0.98 ± 0.006	0.98 ± 0.003	0.96 ± 0.01	0.97 ± 0.01	0.97 ± 0.01	χ^2^ (4) = 1.25, p = 0.86	χ^2^ (2) = 0.95, p = 0.62	χ^2^ (2) = 1.14, p = 0.56	0.12	0.14
Incongruent SCWT (ms)	N	1400.81 ± 88.16	1262.62 ± 89.49	1125.81 ± 79.92	1332.08 ± 66.28	1184.25 ± 60.82	1111.81 ± 55.70	1343.53 ± 85.06	1277.67 ± 84.14	1170.92 ± 65.42	χ^2^ (4) = 9.31, p = 0.053	χ^2^ (2) = 0.67, p = 0.71	**χ**^2^** (2) = 66.02, p < 0.001**	0.28	0.89
Accuracy	Y	0.90 ± 0.02	0.94 ± 0.01	0.96 ± 001	0.86 ± 0.05	0.92 ± 0.02	0.96 ± 0.01	0.87 ± 0.03	0.94 ± 0.02	0.97 ± 0.009	χ^2^ (4) = 5.02, p = 0.28	χ^2^ (2) = 0.51, p = 0.77	**χ**^2^** (2) = 9.28, p = 0.009**	0.93	0.99
Neutral SCWT (ms)	N	1205.30 ± 67.78	1070.25 ± 64.22	1004.92 ± 67.76	1131.26 ± 62.32	1023.18 ± 49.43	961.88 ± 44.23	1184.87 ± 90.16	1119.25 ± 78.72	1025.50 ± 59.09	χ^2^ (4) = 3.62, p = 0.45	χ^2^ (2) = 1.00, p = 0.60	**χ**^2^** (2) = 38.21, p < 0.001**	0.29	0.85
Accuracy	N	0.96 ± 0.006	0.97 ± 0.009	0.98 ± 0.005	0.96 ± 0.01	0.99 ± 0.003	0.98 ± 0.003	0.96 ± 0.01	0.97 ± 0.01	0.98 ± 0.004	χ^2^ (4) = 1.95, p = 0.74	χ^2^ (2) = 0.60, p = 0.73	χ^2^ (2) = 1.22, p = 0.54	0.14	0.18

a: Indicates whether baseline values were included as covariates in the model (Y = included; N = not included). Baseline values were included when marginal or significant group differences were observed at baseline. b: Non-significant in post hoc analyses. ANT: Attention Network Test; No-cue, Center-cue, Double-cue, and Spatial-cue refer to the respective conditions in the ANT (reaction time measures). SCWT: Stroop Color–Word Test; Congruent, Incongruent, and Neutral refer to the respective conditions in the SCWT

No interaction effect was observed in the executive control network. A significant main effect of Time indicated improved efficiency after the intervention and at follow-up (χ^2^ (2)  = 21.15, p < 0.001); post hoc comparisons showed significant improvements both after the intervention (95% CI [− 0.37, − 0.13], p < 0.001) and at follow-up (95% CI [− 0.44, − 0.20], p < 0.001; see Table 2; Fig. 5c).

### Cue conditions

**Fig. 6:**
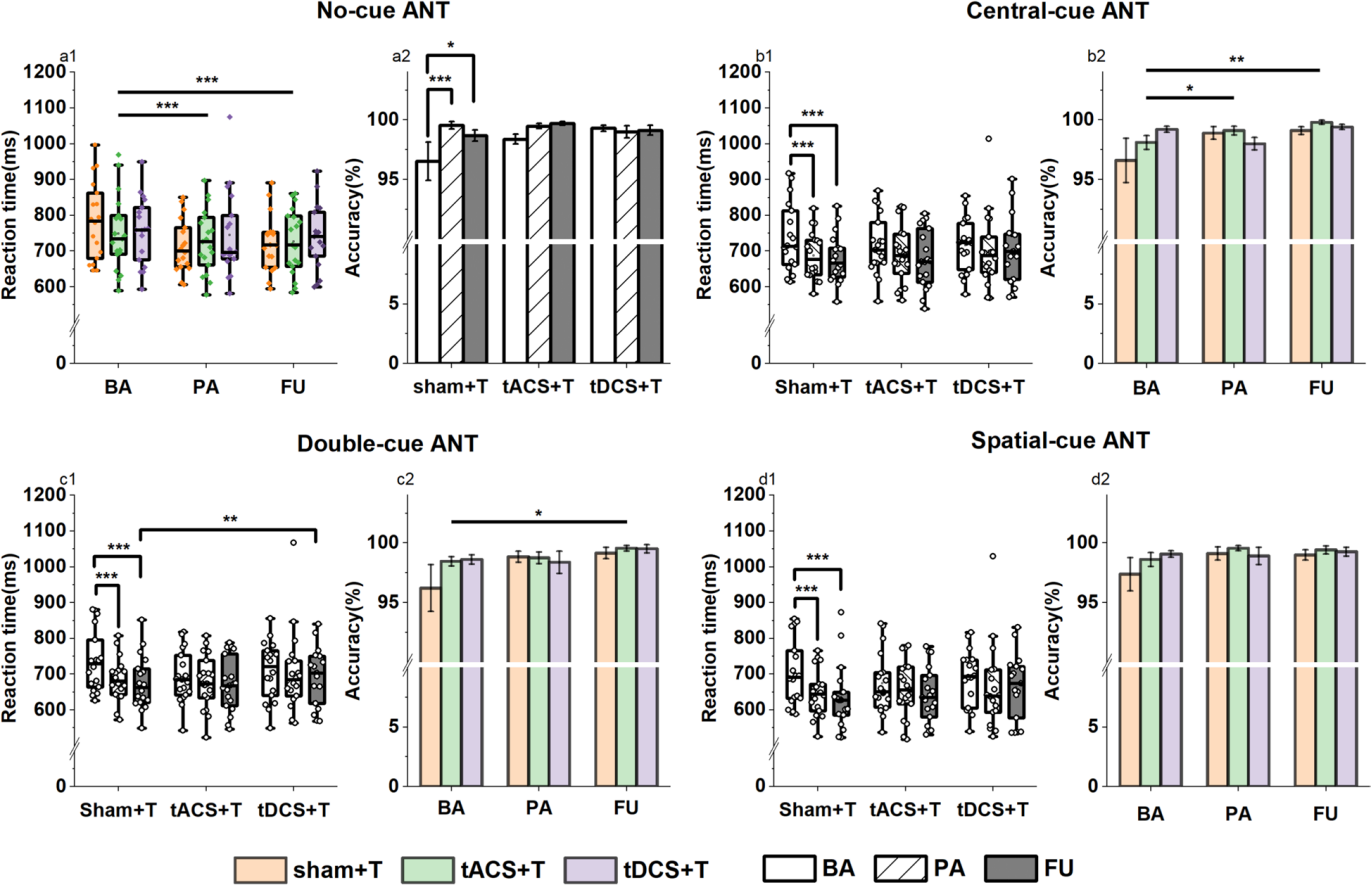
Performance across different ANT conditions. Data are presented as boxplots with overlaid individual participant data points for each group (Sham + T, tACS + T, tDCS + T). The box represents the IQR, the horizontal line indicates the median, and whiskers extend to 1.5 × IQR. Colors represent different groups, whereas different fill patterns indicate assessment time points (BA, PA, FU). Panels labeled “1” (a1–d1) display reaction time (ms), and panels labeled “2” (a2–d2) display accuracy. **a**–**d** Correspond to the No-cue, Center-cue, Double-cue, and Spatial-cue conditions, respectively. For visualization purposes, the x-axis was adapted according to the type of statistical effect: for panels showing a main effect of time, time (BA, PA, FU) is displayed on the x-axis with groups represented by color; for panels showing interaction effects, group is displayed on the x-axis with time represented by different fill patterns. p < 0.05; *p < 0.01; **p < 0.001

No significant interaction was observed for RT under the no-cue condition (χ^2^ (2)  = 9.30, p = 0.053; see Table 2). A significant main effect of Time indicated decreased RT from baseline to post-intervention and follow-up (χ^2^ (2) = 24.25, p < 0.001), with reductions observed from baseline to post-intervention (95% CI [− 0.07, − 0.01], p < 0.001) and from baseline to follow-up (95% CI [− 0.06, − 0.01], p = 0.001; see Table 2; Fig. 6 @a1), without significant group differences.

For accuracy, a significant Group × Time interaction was observed (F (4,151) = 2.54, p = 0.04; Fig. 6 @a2). Post hoc comparisons indicated that the sham + T group showed improved performance from baseline to post-intervention (95% CI [0.01, 0.05], p < 0.001) and from baseline to follow-up (95% CI [0.003, 0.04], p = 0.01; see Table 2).

For the central-cue condition, a significant Group × Time interaction was observed for RT (χ^2^ (4) = 10.28, p = 0.03; Fig. 6 @ b1). Post hoc comparisons indicated that only the sham + T group showed faster RT after the intervention and at follow-up (post-intervention: 95% CI [− 0.11, − 0.02], p < 0.001; follow-up: 95% CI [− 0.12, − 0.04], p < 0.001; see Table 2). For accuracy, a significant main effect of Time was observed, indicating increased accuracy from baseline to post-intervention and follow-up (F (2,151) = 6.30, p = 0.002; post-intervention: 95% CI [0.0006, 0.02], p = 0.03; follow-up: 95% CI [− 0.004, 0.02], p = 0.002; Fig. 6 @ b2), without significant group differences.

Under the double-cue condition, a significant Group × Time interaction was observed for RT (χ^2^ (4) = 13.34, p = 0.01; Fig. 6 @ c1). Post hoc analyses showed that the sham + T group demonstrated faster RT at post-intervention and follow-up compared with baseline (post-intervention: 95% CI [− 0.10, − 0.03], p < 0.001; follow-up: 95% CI [− 0.12, − 0.04], p < 0.001; see Table 2). In addition, the sham + T group showed shorter RT compared to the tDCS + T group at follow-up (95% CI [− 0.11, − 0.02], p = 0.002). For accuracy, a significant main effect of Time indicated improved performance from baseline to follow-up (χ^2^ (4) = 8.86, p = 0.01; 95% CI [0.0008, 0.03], p = 0.03; Fig. 6 @ c2), without significant group differences.

For the spatial-cue condition, a significant Group × Time interaction was observed for RT (χ^2^ (4) = 12.75, p = 0.01; Fig. 6 @ d1). Post hoc comparisons indicated that the sham + T group showed reduced RT after the intervention and at follow-up compared with baseline (post-intervention: 95% CI [− 0.12, − 0.03], p < 0.001; follow-up: 95% CI [− 0.14, − 0.06], p < 0.001; see Table 2), without significant group differences. No significant effects were observed for accuracy (Fig. 6 @ d2).

## Stroop color-word test

**Fig. 7:**
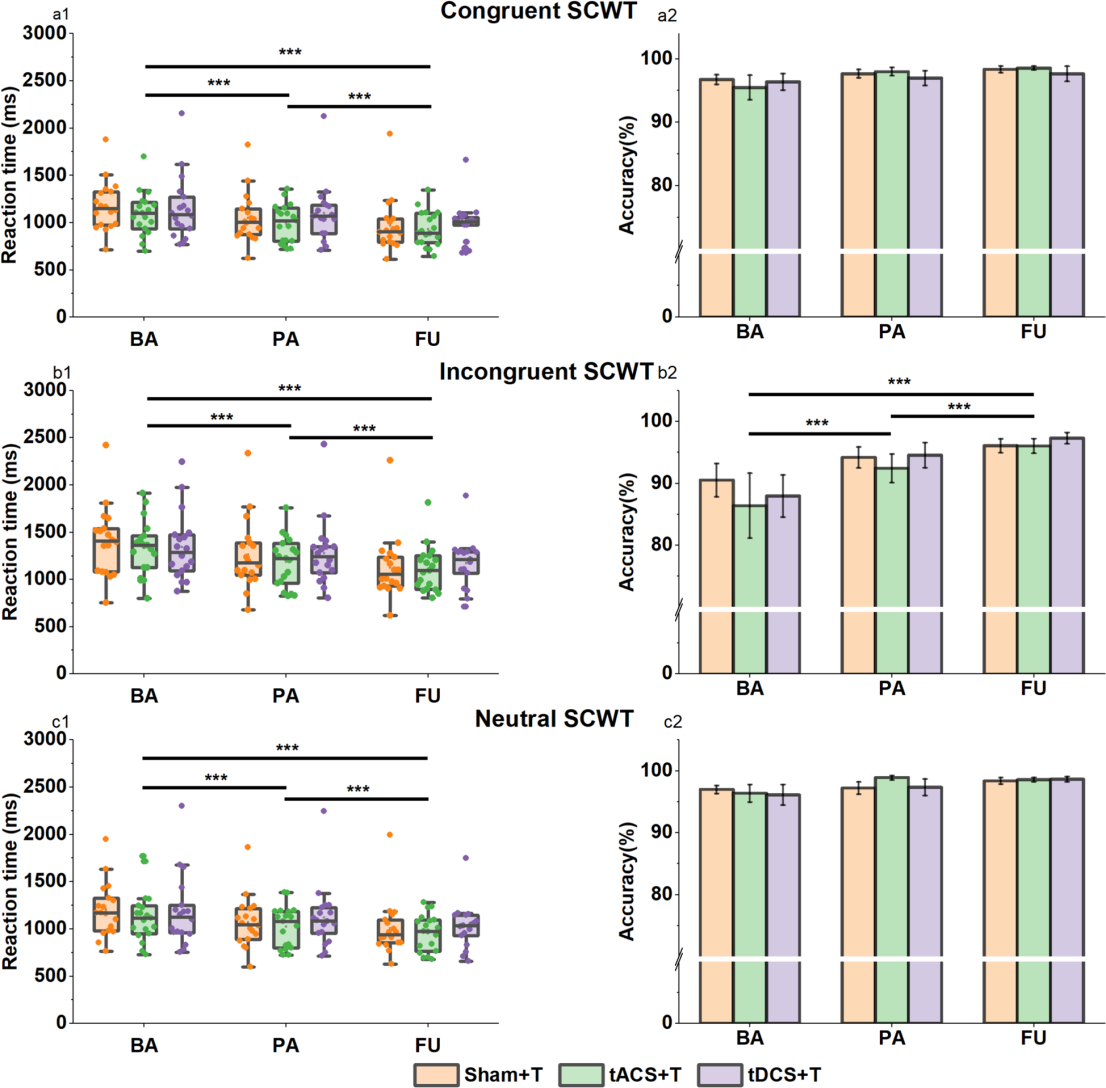
Performance on the Stroop task. Data are presented as boxplots with overlaid individual participant data points for each group (Sham + T, tACS + T, tDCS + T). The box represents the IQR, the horizontal line indicates the median, and whiskers extend to 1.5 × IQR. Panels labeled “1” (a1–c1) display reaction time (ms), whereas panels labeled “2” (a2–c2) display accuracy (%). **a**–**c** correspond to the congruent, incongruent, and neutral conditions, respectively. BA = baseline; PA = post-assessment; FU = follow-up. *: 0.01 < p ≤ 0.05; **: 0.001 < p ≤ 0.01; ***: p ≤ 0.001

In the congruent condition, a significant main effect of Time indicated reduced RT across time (χ^2^ (2) = 45.13, p < 0.001; Fig. 7 @ a1). Post hoc comparisons showed reductions from baseline to post-intervention (95% CI [− 0.12, − 0.04], p < 0.001), from post-intervention to follow-up (95% CI [− 0.11, − 0.03], p < 0.001), and from baseline to follow-up (95% CI [− 0.20, − 0.11], p < 0.001; see Table 2). No alternation of accuracy was found (Fig. 7 @ a2, Table 2).

In the incongruent condition, no significant interaction was observed (χ^2^ (4) = 9.31, p = 0.053). A significant main effect of Time indicated reduced RT across time (χ^2^ (2) = 66.02, p < 0.001; Fig. 7 @ b1). Post hoc comparisons showed reductions from baseline to post-intervention (95% CI [− 0.13, − 0.05], p < 0.001), from baseline to follow-up (95% CI [− 0.20, − 0.13], p < 0.001), and from post-intervention to follow-up (95% CI [− 0.11, − 0.03], p < 0.001; see Table 2). For accuracy, a significant main effect of Time was observed (χ^2^ (2) = 9.28, p = 0.009; Fig. 7 @ b2). Post hoc comparisons indicated improved performance at post-intervention (95% CI [0.33, 1.01], p < 0.001), at follow-up (95% CI [0.81, 1.58], p < 0.001), and from post-intervention to follow-up (95% CI [0.12, 0.90], p < 0.001; see Table 2).

In the neutral condition, a significant main effect of Time indicated reduced RT across time (χ^2^ (2) = 38.21, p < 0.001; Fig. 7 @ c1). Post hoc comparisons showed reductions from baseline to post-intervention (95% CI [− 0.13, − 0.05], p < 0.001), from baseline to follow-up (95% CI [− 0.19, − 0.11], p < 0.001), and from post-intervention to follow-up (95% CI [− 0.10, − 0.02], p = 0.001; see Table 2). No significant effects were observed for accuracy (Fig. 7 @ c2).

### Correlation between attention networks and Stroop Performances

We further investigated the correlations between Stroop performance (congruent, incongruent, neutral) and alerting, orienting, executive control network efficiencies.

Our model did not find significant interaction or any main effects regarding the correlation between the alerting network efficiency and congruent performance (Table 3).

**Table 3.** Interaction and main effects of the correlations between network efficiencies and Stroop Color-Word Test performances

Correlation	Sham + T			tACS + T			tDCS + T			Interaction	Group effect	Time effect	R^2^m	R^2^c
	BA	PA	FU	BA	PA	FU	BA	PA	FU					
ANT-Congruent														
alerting-congruent	− 0.03 ± 0.68	− 1.25 ± 1.25	− 0.69 ± 0.87	0.04 ± 0.60	0.15 ± 0.88	− 0.83 ± 0.74	1.33 ± 1.31	0.54 ± 0.85	− 1.80 ± 1.36	F (4, 111.87) = 0.78, p = 0.54	F (2, 122.22) = 0.29, p = 0.74	F (2, 122.22) = 0.29, p = 0.74	-	-
orienting-congruent	0.48 ± 0.82	− 1.28 ± 0.78	− 2.26 ± 0.67	− 1.65 ± 0.89	0.82 ± 1.08	1.32 ± 1.36	− 0.32 ± 1.13	0.48 ± 1.09	− 1.14 ± 0.95	**F (4, 113.36) = 3.29, p = 0.01, ηp**^**2**^** = 0.10**	F (2, 117.57) = 1.32, p = 0.26	F (2, 113.553) = 0.46, p = 0.63	-	-
executive-congruent	0.28 ± 0.30	0.37 ± 1.05	0.85 ± 1.05	− 0.03 ± 0.83	− 0.48 ± 1.12	− 0.43 ± 0.99	3.77 ± 1.27	− 1.33 ± 1.05	1.10 ± 1.39	F (4, 114.28) = 2.06, p = 0.08	F (2, 128.26) = 1.51, p = 0.31	**F (2, 114.84) = 3.31, p = 0.03, ηp**^2^** = 0.05**	-	-
ANT-Incongruent														
alerting-incongruent	− 0.67 ± 0.76	− 0.97 ± 1.38	− 0.11 ± 0.97	0.43 ± 0.67	0.53 ± 0.97	0.23 ± 0.92	− 0.32 ± 1.46	− 0.14 ± 0.94	− 1.90 ± 1.50	F (4, 111.19) = 0.45, p = 0.76	F (2, 119.13) = 1.08, p = 0.34	F (2, 111.25) = 0.18, p = 0.83	-	-
orienting-incongruent	0.005 ± 0.92	− 1.97 ± 0.88	− 1.99 ± 0.75	− 1.37 ± 0.99	0.26 ± 1.21	1.26 ± 1.52	− 0.43 ± 1.27	0.41 ± 1.23	− 0.93 ± 1.06	F (4, 112.07) = 1.93, p = 0.11	F (2, 115.39) = 1.53, p = 0.22	F (2, 112.21) = 0.02, p = 0.97	-	-
executive-incongruent	0.06 ± 0.33	− 0.10 ± 1.16	− 0.97 ± 1.16	1.04 ± 0.92	0.82 ± 1.25	0.54 ± 1.11	2.63 ± 1.41	− 1.08 ± 1.16	1.99 ± 1.53	F (4, 112.56) = 1.24, p = 0.29	F (2, 123.16) = 1.38, p = 0.25	F (2, 112.97) = 1.52, p = 0.22	-	-
ANT-Neutral														
alerting-neutral	− 0.50 ± 0.72	− 1.49 ± 1.32	− 0.16 ± 0.93	− 0.53 ± 0,64	0.64 ± 0.93	− 0.44 ± 0.79	2.44 ± 1.40	0.48 ± 0.90	− 1.53 ± 1.44	F (4, 111.78) = 1.94, p = 0.11	F (2, 121.76) = 0.69, p = 0.49	F (2, 111.85) = 1.40, p = 0.25	-	-
orienting-neutral	0.71 ± 0,88	− 2.29 ± 0.84	− 2.46 ± 0.71	− 0.64 ± 0.95	0.35 ± 1.15	0.35 ± 1.45	− 0.58 ± 1.21	0.26 ± 1.17	− 1.66 ± 1.01	F (4, 113.22) = 2.26, p = 0.06	F (2, 117.37) = 1.50, p = 0.22	F (2, 113.40) = 1.06, p = 0.34	-	-
executive-neutral	0.08 ± 0.32	0.34 ± 1.11	0.15 ± 1.21	0.16 ± 0.88	0.28 ± 1.20	0.03 ± 1.06	4.85 ± 1.35	− 0.61 ± 1.12	1.01 ± 1.48	F (4, 113.92) = 2.29 p = 0.06	F (2, 127.52) = 1.65, p = 0.19	F (2, 114.45) = 2.75, p = 0.07	-	-

Data are presented as Slopes ± SE, representing the local regression slope of networks (alerting, orienting, and executive control) on SCWT for each Group × Visit. For each time point, networks were mean-centered within each group correspondingly. Positive slopes indicate that higher network efficiency scores (poor efficiency) were associated with longer SCWT reaction times (lower performance), whereas negative slopes indicate that higher network efficiency scores were associated with shorter reaction times

**Fig. 8:**
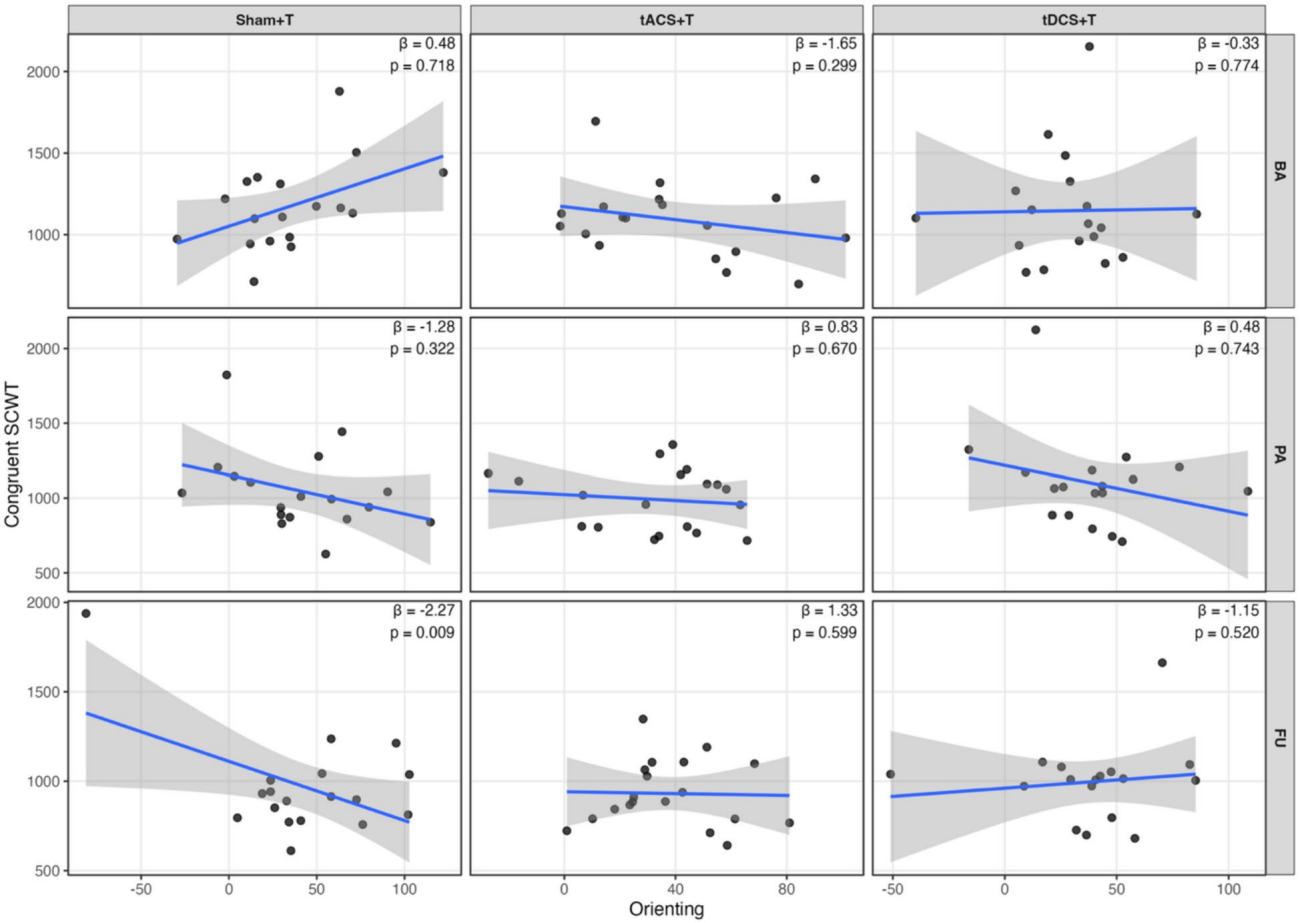
Scatterplots illustrating the association between orienting network efficiency and congruent reaction time (ms) across groups and time points. Each panel represents one group (columns: Sham + T, tACS + T, tDCS + T) and one assessment time (BA = baseline; PA = post-assessment; FU = follow-up). Dots represent individual participants, and solid lines indicate fitted linear regression lines with shaded areas representing 95% confidence intervals. The β coefficients and corresponding p-values are displayed in each panel. A significant negative association was observed in the Sham + T group at follow-up, while no significant associations were found in the other conditions

For the association between orienting network efficiency and congruent performance, a significant three-way interaction was observed (F (4,113.36) = 3.29, p = 0.01, Fig. 8). Simple-slope analyses indicated that orienting network scores were negatively associated with congruent RT in the Sham + T group at follow-up, with a stronger association compared to baseline (95% CI [0.74, 4.76], p = 0.024). No significant slopes were observed in the other groups.

A main Time effect (F (2, 114.84) = 3.32, p = 0.03) of correlation between the executive control network efficiency and congruent performance was revealed. However, post-hoc reported no statistically significant differences after correction.

No significant interaction or main effects were observed for the correlations between ANT network efficiencies and performance under either incongruent or neutral conditions.

## Blinding effect and side effects

The proportion of correct guesses regarding the stimulation condition (34.5%; 19 out of 55 participants: sham + T: n = 6, tACS + T: n = 5, tDCS + T: n = 8) was significantly below the expected 50% chance level (χ^2^ = 5.25, p = 0.02). This result suggests that participants were unable to accurately discern which type of stimulation they had received, confirming that the blinding procedure was effectively maintained.

None of the included participants discontinued the stimulation because of side or adverse events related to the combined intervention or needed a medical intervention following the stimulation. The most frequently reported experience was a mild tingling sensation, which occurred in 33.3% of participants in the sham + T group, 42.1% in the tACS + T group, and 27.7% in the tDCS + T group. Other mild and transient sensations included dizziness (11.1%, 5.3%, and 11.1%), nervousness (11.1%, 10.5%, and 27.7%), headache (11.1%, 15.8%, and 11.1%), and fatigue (16.7%, 15.8%, and 16.7%) across the respective groups. Overall, these sensations were mild, transient, and comparable among the groups. We did not observe any skin redness in any of the participants.

## Discussion

In this randomized, triple-blind, sham-controlled study, the effects of multi-session concurrent left-DLPFC tDCS / theta-tACS and task training on cognitive performance in healthy older adults were assessed and compared. Participants completed ten 20-min sessions and cognitive functions were assessed before, after, and four weeks after the intervention using ANT and SCWT.

We hypothesized that the group receiving theta-tACS paired with training would show superior effects on improving cognitive attention and inhibition, followed by tDCS, and then sham. It has been reported that a single session of tACS could induce moderate effects on memory enhancement, while comparable to multi-session tDCS [74]. Contrary to our hypotheses, only sham + T group showed significantly improvements with long-term efficacy in ANT. For SCWT, improved executive function after intervention independent of group was observed. Notably, we did not find any synergistic effect of tDCS or tACS in combined methods, when compared with sham stimulation in all conditions. The enhancements in cognitive function across all groups post-intervention may be primarily due to the repeated task training rather than additive effects of tDCS or tACS.

Among the studies that have explored the combined effects in cognition, previous work has yielded inconsistent conclusions about the relative contribution of real stimulation versus training in combined protocols. Combining 12 60-min sessions of daily computerized cognitive training and 20-min theta-tACS targeting the left-DLPFC in patients with MCI demonstrated improved attention and executive function measured via oddball task in only the active stimulation group [75]. Another study applied single 20-min session combined treadmill walking and anodal high-definition tDCS targeting both the primary motor cortex and the left-DLPFC in the healthy elderly, only participants in real stimulation group demonstrated improved SCWT performance [76]. However, Martin et al., [77] applied 15 30-min sessions of concurrent tDCS targeting left-DLPFC and working memory training in amnestic-MCI patients, results revealed that both the active and sham tDCS groups showed significant improvements in memory, while no significant differences were observed between groups. The inconsistencies can be attributed to multiple factors, such as various montages, stimulation sessions, difference between programs, as well as targeted populations.

We did not observe additional benefits of active stimulation compared with sham stimulation. Notably, enhanced performance was observed in the sham + T group under the central-cue, double-cue, and spatial-cue conditions of the ANT. However, these findings should be interpreted with caution. As the present study did not include a no-intervention or training-only control group, the relative contributions of task training and potential placebo or expectation effects associated with sham stimulation cannot be clearly disentangled [78]. Importantly, sham stimulation is not an inert condition, as it can induce expectations of receiving an effective intervention. Such expectations may modulate behavioral performance through non-specific mechanisms, including increased attention, motivation, and task engagement [79, 80]. In the context of non-invasive brain stimulation, these expectation-driven effects have been shown to influence behavioral outcomes and may partially account for improvements observed under sham conditions [81]. Therefore, the performance enhancements observed in the sham + T group in the present study may not solely reflect task training effects, but could also be influenced by placebo-related mechanisms.

One possible explanation for the limited improvements observed in the active stimulation groups is the relatively high baseline performance. For instance, the mean baseline no-cue accuracy for the tACS + T and tDCS + T groups was already at 98% and 99%. Similarly, the mean RTs in the double-cue condition were 691.38 ms and 708.32 ms at baseline and changed only slightly to 679.16 ms and 705.92 ms after the intervention (Table 2). This suggests a potential ceiling effect, which may have limited further performance gains.

Another possibility is the add-on effects of tES was overshadowed by the training effects and remain undetectable. Similar results were reported in emerging studies [82]. Lau et al.[83] concurrently applied 15 sessions of cognitive-related video-game trainings with anodal tDCS of the left-DLPFC at 2 mA in MCI patients. Results revealed better working memory performance and executive function in both real and sham stimulation groups after intervention without statistical differences. Diedrich et al. [84] reported enhanced memory function in both active and sham stimulation groups after 16 20-min sessions of computerized cognitive training paired with theta-gamma tACS targeting bilateral DLPFC in healthy older adults.

Additionally, another possible explanation could be the potential cancellation between the additional modulatory effects of stimulation and the already elevated neural activation induced by task training. Evidence from neurophysiological studies further indicated that neural plasticity including long-term potentiation / depression is critically depend on intracellular Ca^2^⁺ levels [85]. When Ca^2^⁺ is already elevated during motor activation, additional stimulation-induced depolarization may no longer enhance plasticity, or even tip the facilitation process [54, 85, 86]. Previous work has shown increased cortical excitability at rest, but reduced excitability during concurrent right-hand tasks when anodal tDCS [53, 87] or 140-Hz tACS [54] targeting the left primary motor cortex. Taken together, these findings indicate that the effects of external stimulation are highly state-dependent. Moreover, other factors including participants’ baseline neural state, population characteristics, number of intervention sessions, stimulation parameters, and intervention duration warrant further investigation and careful consideration in future study designs [88].

An interesting result we observed is that improvements in Stroop performance were evident across all groups, but these behavioral gains were not accompanied by parallel changes across the three attentional networks. Only the executive control network showed a reliable improvement over time, suggesting that executive control is particularly sensitive to practice and cognitive training and in line with previous study [89, 90]. Nevertheless, the enhanced executive control efficiency did not correlate with improved Stroop performance (executive function), and alerting remained unchanged. Previous study suggested that basic arousal mechanisms played a limited role in Stroop tasks [91]. As a result, the overall improvement in Stroop performance unlikely to be induced from the strengthening of the attentional system itself. Furthermore, in the sham + T group, the orienting network efficiency demonstrated significant negative correlation to SCWT within the congruent condition. More specifically, at follow-up, participants in the sham + T group who have higher orienting network score (representing actual lower efficiency) tended to respond faster in the congruent condition. Although this negative association may seem counterintuitive, previous work demonstrated that orienting contributes less to performance once a task becomes familiar and the attentional demands shift toward more automatic processing modes [92, 93]. Reduced reliance on orienting may therefore reflect a gradual adjustment in attentional strategy, in which participants no longer need to direct spatial attention deliberately to solve simple trials. The absence of similar associations in the combined tES groups may suggesting that neuromodulation altered the redistributed of attention resource. Even though no significant group differences were observed after the intervention, comparable performance can arise from distinct underlying cognitive pathways [94]. Taken together, these findings suggest that the observed improvements in Stroop performance may reflect a shift in the coordination of attentional resources rather than direct enhancement of a specific attentional subsystem.

## Limitation

Our findings should be interpreted in the light of the study limitations. The lack of a control group without any intervention prevents us to draw definitive conclusions regarding the effects of the task training. Furthermore, integrating electroencephalography and functional magnetic resonance imaging would allow a more comprehensive investigation of brain network dynamics and cognitive resource allocation during cognitive process by e.g., combining event-related time–frequency analysis with high spatial precision [95]. Future research should also consider variations in participant characteristics (e.g., identical training program may demonstrate higher efficiency in patients with motor or cognitive deficits [29]), electrode montages, and intervention durations to enhance generalizability and refine the understanding of tACS / tDCS–training interactions. A further limitation is that participants’ expectations regarding the intervention were not formally assessed. Although the blinding results suggested comparable expectations across groups, expectation effects may still have influenced the outcomes, as previous studies have highlighted the role of participants’ beliefs in non-invasive brain stimulation [78].

## Conclusion

This study demonstrated that ten 20-min sessions of combined tES and task training did not produce superior effects of enhancement on cognitive function in healthy older adults. The observed improvements were mainly attributed to the training program rather than the stimulation itself, indicating the dominant role of task training within combined intervention approaches. The absence of additional tES effects may be explained by a potential cancellation between the modulatory influence of stimulation and the already elevated neural activation induced by training. While the intervention enhanced executive control and general task performance over time, only orienting engaged in a specific linkage with congruent Stroop processing, underscoring a heterogeneous pattern of transfer across attentional networks.

## Supplementary Information

Additional file1 (DOCX 19 KB)

## Data Availability

The datasets used and/or analysed during the current study are available from the corresponding author on reasonable request.

## Abbreviations

tES: Transcranial electrical stimulation
RT: Reaction time
tDCS: Transcranial direct current stimulation
tACS: Transcranial alternating current stimulation
sham + T: Concurrent sham stimulation combined with training group
tACS + T: Concurrent tACS combined with training group
tDCS + T: Concurrent tDCS combined with training group
ANT: Attention network test
SCWT: Stroop Color-Word Test
RT: Reaction time
DLPFC: Dorsolateral prefrontal cortex
MCI: Mild cognitive impairment
MoCA: Montreal Cognitive Assessment
SE: Standard error of the mean
BA: Baseline assessment
PA: Post-assessment
FU: Follow-up assessment, four weeks after the last intervention session
LMM: Linear mixed-effects models
GLMM: Generalized linear mixed-effects models
ηp^2^: Partial eta squared
R^2^m: Marginal R^2^
R^2^c: Conditional R^2^
