# Supplementary material for: Training-induced cognitive coupling emerges under sham but not active transcranial electrical stimulation in older adults: a triple-blind, randomized, sham-controlled study
Source: J Neuroeng Rehabil. 2026 Jul 14;23:222. doi: 10.1186/s12984-026-02077-5 (PMC13371604; doi:10.1186/s12984-026-02077-5)
Supplement: Supplementary file 1 — Additional file1 (DOCX 19 KB) [file 12984_2026_2077_MOESM1_ESM.docx]

**Supplementary material**

All mixed-effects analyses were conducted in R (version 4.5.1) and RStudio. Linear mixed-effects models were fitted using the lme4 (version 1.1-37) and lmerTest (version 3.1-3) packages. Generalized linear mixed-effects models were fitted using glmmTMB (version 1.1.11). Post-hoc comparisons were conducted using emmeans (version 1.11.2). Effect sizes and model diagnostics were obtained using effectsize (version 1.0.1), MuMIn (version 1.48.11), performance (version 0.15.0), and car (version 3.1-3).

Supplementary Table S1. Mixed-effects models used for statistical analysis.

| Parameter | ModelType | Family | Link |
| --- | --- | --- | --- |
| Alerting | LMM | gaussian | identity |
| Orienting | GLMM | Gamma | log |
| Executive control network | GLMM | Gamma | log |
| No-cue ANT | GLMM | Gamma | log |
| No-cue Accuracy | LMM | gaussian | identity |
| Central-cue ANT | GLMM | Gamma | log |
| Central-cue Accuracy | LMM | gaussian | identity |
| Double-cue ANT | GLMM | Gamma | log |
| Double-cue Accuracy | GLMM | Gamma | log |
| Spatial-cue ANT | GLMM | Gamma | log |
| Spatial-cue Accuracy | GLMM | Gamma | log |
| Congruent SCWT | GLMM | Gamma | log |
| Congruent SCWT Accuracy | GLMM | Gamma | log |
| Incongruent SCWT | GLMM | Gamma | log |
| Incongruent SCWT Accuracy | GLMM | Gamma | log |
| Neutral SCWT | GLMM | Gamma | log |
| Neutral SCWT Accuracy | GLMM | Gamma | log |
| Alerting-congruent | LMM | gaussian | identity |
| Orienting-congruent | LMM | gaussian | identity |
| Executive-congruent | LMM | gaussian | identity |
| Alerting-incongruent | LMM | gaussian | identity |
| Orienting-incongruent | LMM | gaussian | identity |
| Executive-incongruent | LMM | gaussian | identity |
| Alerting-neutral | LMM | gaussian | identity |
| Orienting-neutral | LMM | gaussian | identity |
| Executive-neutral | LMM | gaussian | identity |
